# The Adnectin CT-322 is a novel VEGF receptor 2 inhibitor that decreases tumor burden in an orthotopic mouse model of pancreatic cancer

**DOI:** 10.1186/1471-2407-8-352

**Published:** 2008-11-27

**Authors:** Sean P Dineen, Laura A Sullivan, Adam W Beck, Andrew F Miller, Juliet G Carbon, Roni Mamluk, Henry Wong, Rolf A Brekken

**Affiliations:** 1 Division of Surgical Oncology, Department of Surgery, University of Texas Southwestern Medical School, Dallas, TX 75390-8593, USA; 2 Hamon Center for Therapeutic Oncology Research, University of Texas Southwestern Medical School, Dallas, TX 75390-8593, USA; 3 Adnexus, a Bristol-Myers Squibb Company, Waltham, MA 02453, USA; 4 Department of Pharmacology, University of Texas Southwestern Medical School, Dallas, TX 75390-8593, USA

## Abstract

**Background:**

Pancreatic cancer continues to have a 5-year survival of less than 5%. Therefore, more effective therapies are necessary to improve prognosis in this disease. Angiogenesis is required for tumor growth, and subsequently, mediators of angiogenesis are attractive targets for therapy. Vascular endothelial growth factor (VEGF) is a well-characterized mediator of tumor angiogenesis that functions primarily by binding and activating VEGF receptor 2 (VEGFR2). In this study, we evaluate the use of CT-322, a novel biologic (Adnectin). This small protein is based on a human fibronectin domain and has beneficial properties in that it is fully human, stable, and is produced in bacteria. CT-322 binds to and inhibits activation of VEGFR2.

**Methods:**

The efficacy of CT-322 was evaluated *in vivo *using two orthotopic pancreatic tumor models. The first model was a human tumor xenograft where MiaPaCa-2 cells were injected into the tail of the pancreas of nude mice. The second model was a syngeneic tumor using Pan02 cells injected into pancreas of C57BL/6J mice. In both models, therapy was initiated once primary tumors were established. Mice bearing MiaPaCa-2 tumors were treated with vehicle or CT-322 alone. Gemcitabine alone or in combination with CT-322 was added to the treatment regimen of mice bearing Pan02 tumors. Therapy was given twice a week for six weeks, after which the animals were sacrificed and evaluated (grossly and histologically) for primary and metastatic tumor burden. Primary tumors were also evaluated by immunohistochemistry for the level of apoptosis (TUNEL), microvessel density (MECA-32), and VEGF-activated blood vessels (Gv39M).

**Results:**

Treatment with CT-322 was effective at preventing pancreatic tumor growth and metastasis in orthotopic xenograft and syngeneic models of pancreatic cancer. Additionally, CT-322 treatment increased apoptosis, reduced microvessel density and reduced the number of VEGF-activated blood vessels in tumors. Finally, CT-322, in combination with gemcitabine was safe and effective at controlling the growth of syngeneic pancreatic tumors in immunocompetent mice.

**Conclusion:**

We conclude that CT-322 is an effective anti-VEGFR2 agent and that further investigation of CT-322 for the treatment of pancreatic cancer is warranted.

## Background

Pancreatic cancer continues to carry a poor prognosis, with a 5-year survival rate of approximately five percent [1]. As patients typically present at an advanced stage, new chemotherapeutic strategies are necessary to improve the dismal prognosis associated with this disease. Angiogenesis is a hallmark of cancer [2] and is required for cancer growth beyond 1–2 mm^3 ^[2,3]. Vascular endothelial growth factor A (VEGF) is the best characterized member of the VEGF family of growth factors. VEGF is a potent angiogenic factor expressed during development and in tumors [4,5]. The effects of VEGF are mediated by binding to one of its two receptors VEGF receptor 1 or 2 (VEGFR1, VEGFR2) [4,6]. Tumor angiogenesis is driven primarily by VEGF:VEGFR2 interaction [5,6]. The effect of VEGFR1 activation is less understood, but is thought to be involved in macrophage chemotaxis [5-7].

The complexity of the VEGF pathway allows for multiple targets for inhibiting tumor angiogenesis [5,8]. For example, bevacizumab (Avastin^®^, Genentech, Inc., South San Francisco, CA) is a monoclonal antibody to human VEGF which binds VEGF and blocks its interaction with both VEGFR1 and VEGFR2 [9]. Bevacizumab has been shown to be effective in combination with chemotherapy for the treatment of metastatic colorectal cancer and non-small cell lung cancer [10,11]. Receptor tyrosine kinase inhibitors have also been developed which inhibit the VEGF receptors [5,8]. These small molecules penetrate into cells and, unlike antibodies, inhibit multiple members of the VEGF receptor family. This broad spectrum of inhibition may lead to different side effect profiles from monoclonal antibodies [5].

There are a variety of proteins being developed as new biologic drugs beyond the traditional biologic class of monoclonal antibodies [12]. Adnectins are a new class of targeted biologics among the most advanced of such proteins. Adnectins are well-suited to pharmaceutical discovery and development, based on preclinical data [13,14]. These small proteins are derived from the 10th type III domain of human fibronectin, an extracellular protein that is abundant in human serum and the extracellular matrix, and naturally binds to other proteins [13,14]. By changing the amino acid sequence of three targeting loops clustered at one end of the protein, an Adnectin can be designed to bind to a specific disease target, such as a receptor, ligand or protein with nanomolar or picomolar affinity, and potency and specificity comparable to or better than antibodies. One such Adnectin has been developed that binds to VEGFR2. This construct, CT-322, has been shown previously to block the activity of murine and human VEGFR2 in vitro [13].

In the present study, we were interested in whether this novel compound would block tumor angiogenesis and subsequent growth in an orthotopic model of pancreatic cancer. In the following experiments, we demonstrate that CT-322 is effective at treating pancreatic tumors in two animal models, that CT-322 blocks tumor angiogenesis and that treatment with CT-322 induces tumor destruction.

## Methods

### Cell Lines and Culture

The human pancreatic carcinoma cell line MiaPaCa-2 (ATCC, Manassas, VA) and the murine cell line Pan02 also known as Panc02 (NCI, Frederick, MD) were maintained at 37°C in a mixture of 5% CO_2 _and 95% air in Dulbecco's minimal essential medium (DMEM; Invitrogen, Carlsbad, CA) supplemented with 10% fetal calf serum (Gimini Bio-Products, Woodland, CA). Cell suspensions for tumor cell injection (TCI) were made by removing cells from tissue culture flask with 0.2% trypsin. The reaction was stopped with serum-containing DMEM. Cells underwent centrifugation at 230 × *g *for 5 min and the cell pellet was resuspended to the appropriate concentration in PBS. Cell suspensions were used only if viability exceeded 95% by trypan blue exclusion assay.

### Animal Models

Athymic female nude mice were purchased from Charles River (Wilmington, MA). Female C57BL/6J wild-type mice were purchased from Jackson Laboratories (Bar Harbor, MA). Animals were housed with ad libitum access to food and water in a pathogen free facility. All procedures were performed in accordance with a protocol approved by the Institutional Animal Care and Use Committee of UT Southwestern (Dallas, TX).

Orthotopic tumors were established by injection of cells directly into the pancreas as previously described [15]. Briefly, animals were anesthetized using 2–4% isofluorane. The abdominal wall and peritoneum were opened and the spleen and the tail of the pancreas were identified and externalized through the wound. Tumor cells were injected under the capsule the pancreas using a 30-gauge needle (2.5 × 10^5 ^Mia-PaCa-2 cells, 4 × 10^5 ^Pan02 cells, in 50 μl of PBS). The incision was closed with a 5-0 prolene suture.

### Treatment

In the xenograft model (MiaPaCa-2) therapy began 10 days after tumor cell injection (TCI). Therapy groups consisted of control (vehicle provided by Adnexus, Waltham, MA) and CT-322 (30 mg/kg, provided by Adnexus) in the xenograft model. In the syngeneic model (Pan02) a gemcitabine (3.5 mg/mouse) group and a combined CT-322 + gemcitabine group were added. Therapy was given twice weekly by intraperitoneal (i.p.) injection and was continued for six weeks. Animals were sacrificed at this time as control mice developed lethargy and failure to properly groom.

### Immunohistochemistry

Tissue sections were either snap frozen in liquid nitrogen or fixed in formalin (Sigma, St Louis, MO) and paraffin embedded. Hematoxylin and eosin (H&E) staining was done by the Molecular Pathology Core at UT Southwestern (Dallas, TX). Paraffin embedded sections were first de-paraffinized by heating to 60°C followed by xylene immersion. Sections were re-hydrated with sequential ethanol submersion. Non-specific binding was blocked with 20% Aquablock (East Coast Biologics, North Berwick, ME) for 30 minutes. Primary antibody (MECA-32 [16], Gv39M [17]) was added for one hour at room temperature or overnight at 4°C and subsequently developed with the appropriate secondary antibodies (Jackson Immunoresearch Laboratories, West Grove, PA). TUNEL analysis was done per manufacturer's recommendations (Promega, Madison, WI). Immunofluorescent sections were quantified using Nikon Elements imaging software (Melville, NY).

### Statistics

Data was collected into a spreadsheet program (Microsoft Excel) and then imported into GraphPad (GraphPad Prism version 4.00 for Windows; GraphPad Software, San Diego CA, ). Differences between means of two groups were calculated using a Mann-Whitney test; differences between three or more groups were determined with non-parametric one-way ANOVA (Kruskal-Wallis test) with Dunn's post test for multiple comparisons. Statistical significance was established as p < 0.05.

## Results

### CT-322 inhibits the growth of orthotopic MiaPaCa-2 pancreatic tumors

**Figure 1:**
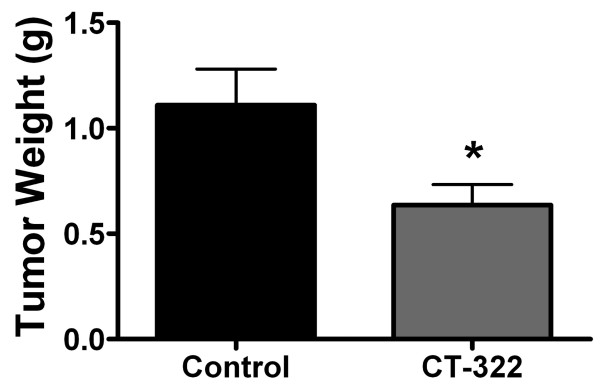
**CT-322 reduces tumor size in a xenograft model of pancreatic cancer**. Human MiaPaCa-2 tumors were established in the tail of the pancreas in athymic nude mice. Therapy was initiated 10 days after tumor cell injection. Treatment with the anti-VEGFR2 Adnectin CT-322 (30 mg/kg) was given by i.p. injection twice weekly for 6 weeks (until sacrifice). Mean (+/- SEM) tumor weight at the time of sacrifice is displayed. CT-322 significantly reduced tumor size compared to control treated animals (Control 1.11 ± 0.17 g, CT-322 0.64 ± .010 g; n = 9 animals per group. *p < 0.05).

To test whether direct inhibition of VEGFR2 with CT-322 inhibits pancreatic tumor growth, we used an orthotopic model of pancreatic cancer. In this model, MiaPaCa-2 human pancreatic cancer cells were injected into the tail of the pancreas of nude mice and tumors were allowed to develop prior to treatment [15]. Therapy consisted of vehicle control or CT-322 given twice weekly and was initiated ten days after tumor cell injection. At sacrifice, tumor weights were measured. CT-322 reduced pancreatic tumor growth by approximately 50% in this model (Fig 1). In this model CT-322 did not reduce metastatic events and the incidence of metastasis was the same (8/9, 88%) in the control and CT-322 treated animals and the mean number of metastases was similar in both groups (1.7, CT-322; 2.1, control).

### CT-322 is effective in combination with gemcitabine in a syngeneic model of pancreatic cancer

**Figure 2:**
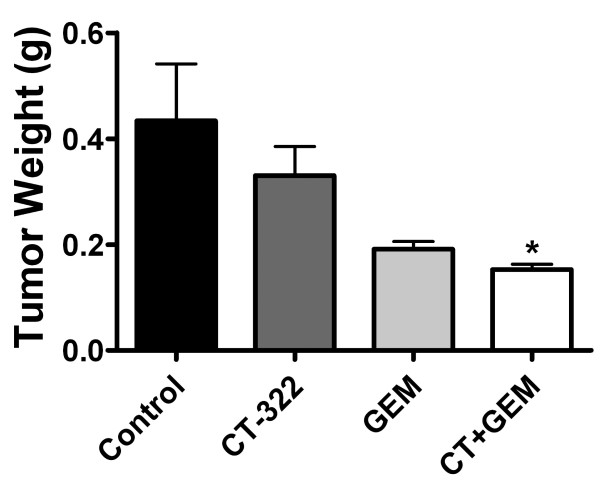
**Tumor size is reduced after CT-322 treatment in a syngeneic model of pancreatic cancer**. Orthotopic murine Pan02 tumors were established in C57BL/6J wild-type mice. Treatment twice weekly i.p. injections of CT-322 (30 mg/kg), gemcitabine (3.5 mg/animal/injection) (GEM), or CT-322 (CT) + GEM was started 7 days after tumor cell injection and continued for 6 weeks, at which point animals were sacrificed. Mean (+/- SEM) tumor weight at the time of sacrifice is displayed. The combination treatment of CT-322 and GEM significantly inhibited tumor growth (control 0.44 ± 0.11 g, 0.153 ± 0.01 g; n = 8/group except CT+GEM n = 10; p < 0.05).

One advantage of CT-322 compared to human monoclonal antibodies specific for VEGF or VEGFR2 is the fact that CT-322 binds to both murine and human VEGFR2. This allows for testing in a syngeneic model using immunocompetent animals. Additionally in this experiment, we tested the combination of CT-322 and gemcitabine, the standard chemotherapy for pancreatic cancer. Tumors were established in C57BL/6J mice using the murine pancreatic carcinoma cell line Pan02. Therapy was started one week later and continued for 6 weeks. The combination of CT-322 and gemcitabine was an effective therapeutic regimen, reducing tumor growth by >50% (Fig 2). Both CT-322 and gemcitabine alone decreased tumor growth, but combination therapy had a greater effect and was the only treatment group to reach statistical significance compared to control-treated animals. Metastatic disease was assessed by visual inspection of abdominal organs, lymph nodes and lungs. In this model all treatment groups showed decreased metastatic burden compared to control (Table 1).

**Table 1:** Effect of therapy on metastatic burden in mice bearing Pan02 tumors

**GROUP**	**METASTASIS**	
	***Number***	***Incidence (%)***

Control	14	6/8 (75)
CT	0	0/8 (0)
GEM	0	0/8 (0)
CT+ GEM	0	0/10 (0)

Metastatic events in mice bearing orthotopic Pan02 tumors were evaluated by visual inspection of the liver, lungs, and entire gastrointestinal tract at the time of sacrifice.

### CT-322 reduces microvessel density

**Figure 3:**
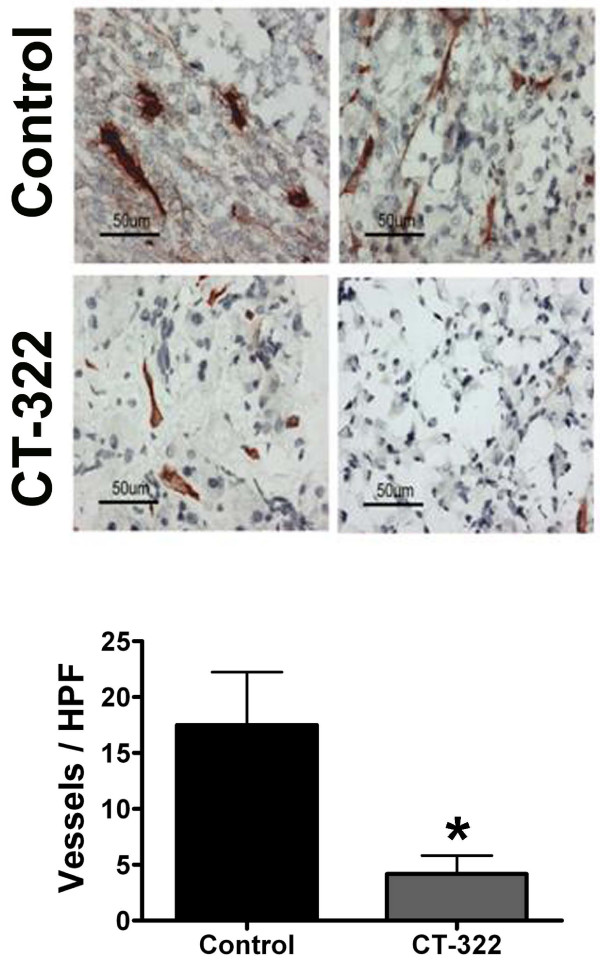
**CT-322 reduces microvessel density in pancreatic cancer xenografts**. MiaPaCa-2 tumor sections from animals treated with CT-322 or control were analyzed for microvessel density by immunohistochemistry using MECA-32. The mean (+/- SEM) number of blood vessels/high power field (total magnification, 400×) is displayed. The number of vessels in tumors from CT-322 treated animals was reduced compared to control (4.2 ± 4.7 versus 17.5 ± 4.7; 3 tumors per group, 3–5 high power fields per tumor; p = 0.001). Two representative pictures from each group are shown.

**Figure 4:**
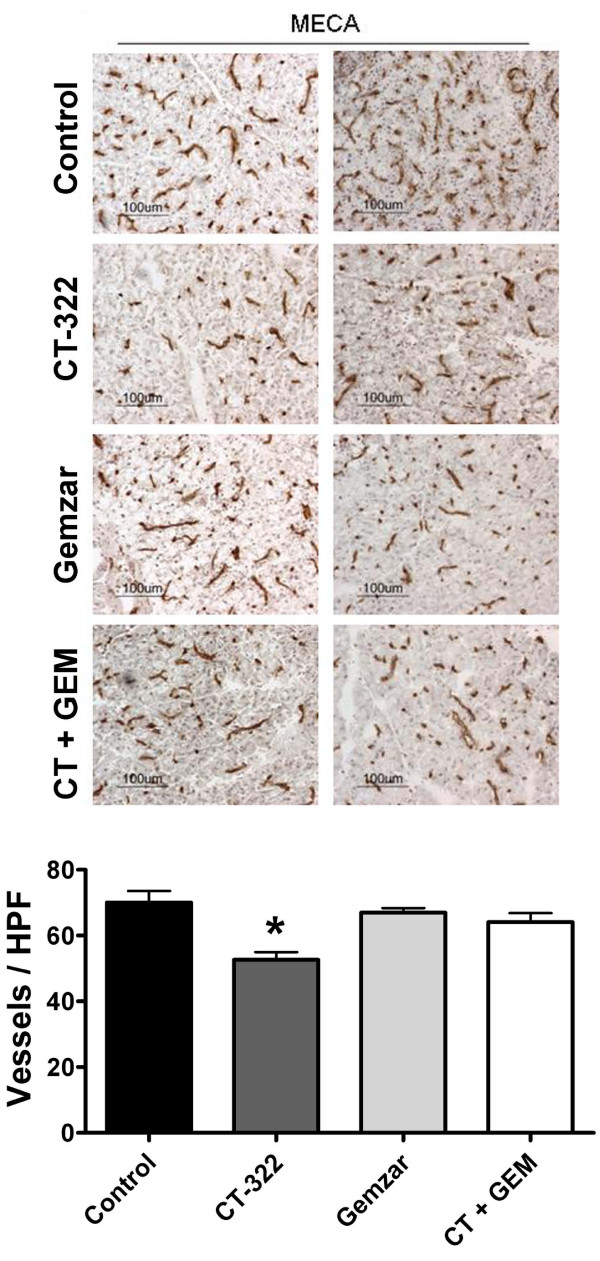
**VEGFR2 inhibition with CT-322 reduces microvessel density in a syngeneic model of pancreatic cancer**. Frozen sections of tumors from mice treated with vehicle alone (Control), CT-322, gemcitabine (GEM), or the combination of CT-322 + gemcitabine were evaluated for microvessel density by immunohistochemistry using MECA-32. Representative images of MECA-32 immunohistochemistry are displayed along with the mean (+/- SEM) number of blood vessels/field (total magnification, 200×). Microvessel density in Pan02 tumors from mice treated with CT-322 monotherapy was reduced compared to tumors from control-treated animals (control, 70.0 ± 3.6; CT-322, 52.6 ± 2.3; 3 tumors per group, at least 5 fields per tumor; *, p < 0.001 vs. control). Two representative pictures from each group are shown.

VEGF inhibition is thought to exert anti-tumor effects in part by inhibiting the development of blood vessels in the tumor. To assess the effect of CT-322 on tumor angiogenesis, sections of tumor were analyzed by immunohistochemistry with MECA-32, an antibody specific for mouse endothelial cells. Treatment with CT-322 reduced tumor microvessel density significantly in the MiaPaCa-2 xenograft model (Fig 3) and the Pan02 syngeneic model (Fig 4).

**Figure 5:**
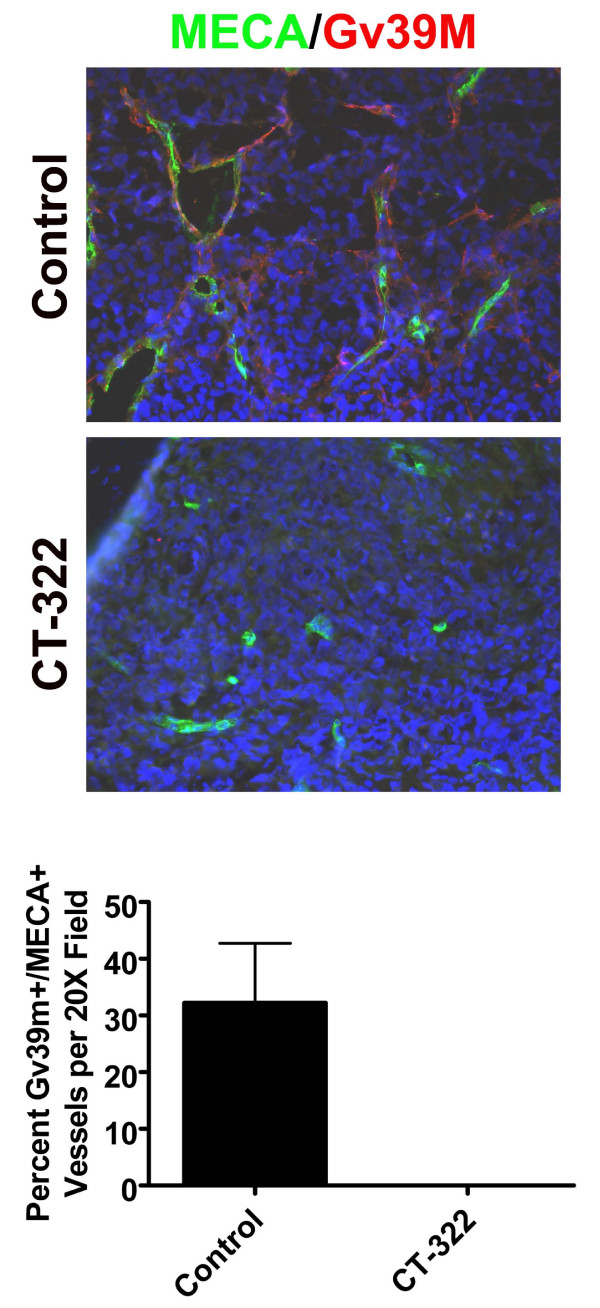
**VEGF-activated blood vessels are reduced after CT-322 treatment**. Immunohistochemistry using Gv39M, an antibody recognizing VEGF-activated blood vessels, and MECA-32 was performed on frozen sections of MiaPaCa-2 tumors from animals treated with control or CT-322. The percentage of MECA-32-positive blood vessels that were stained by Gv39M is displayed. Representative images of co-localization of MECA-32 (green) and Gv39M (red) is shown, magnification 200×. This represents two tumors per group, 8–10 high power fields, p < 0.05.

Furthermore, CT-322 also decreased the level of VEGF-activated blood vessels. VEGF binding to VEGFR2 mediates the activation of endothelial cells and induces angiogenesis [18]. An antibody that recognizes VEGF bound to VEGFR (Gv39M) [17,19] was used to assess whether CT-322 had an effect in vivo on the activation state of blood vessels in MiaPaCa-2 tumors. As shown in figure 5A, there were no blood vessels that were Gv39M positive following CT-322 treatment, whereas approximately 30% of vessels in the control treated animals were positive for a standard endothelial cell marker (MECA-32) and Gv39M (Fig 5B).

### Increased tumor destruction following CT-322 treatment

**Figure 6:**
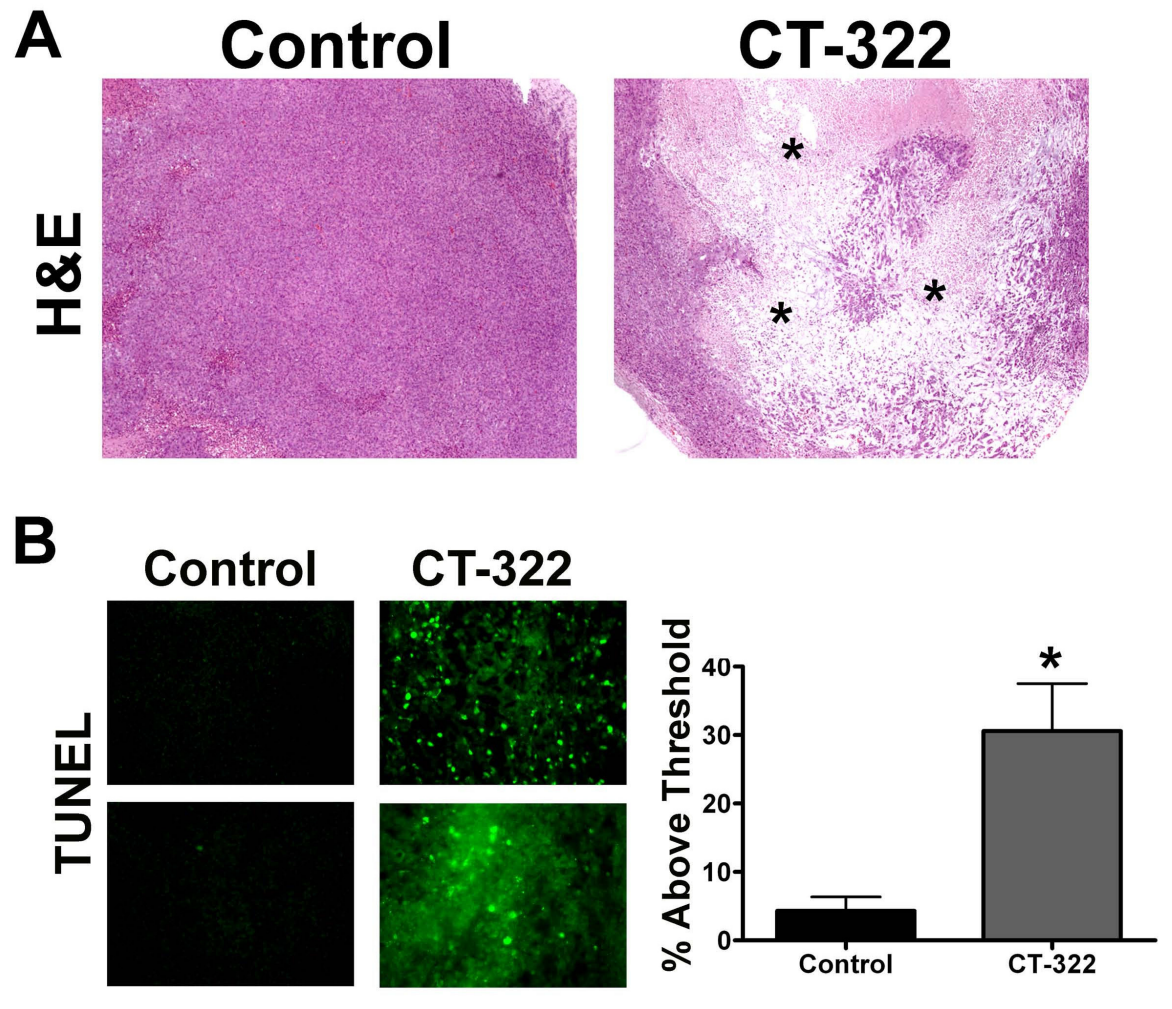
**CT-322 causes tumor destruction**. A) Sections of MiaPaCa-2 tumors were fixed in formalin and paraffin embedded. H&E staining revealed homogenous histology in tumors from the control treated animals. In contrast tumors from CT-322 treated animals showed large areas of tumor destruction (*). Representative images from each group are shown. B) Formalin fixed paraffin embedded MiaPaCa-2 tumor sections from animals treated with control or CT-322 were stained for TUNEL activity. The mean (+/- SEM) % of pixels above threshold is displayed. Treatment with CT-322 increased the number of TUNEL-positive apoptotic cells per tumor section (control 4.3 ± 2.03, CT-322 30.6 ± 6.9). Images were analyzed using Nikon Elements imaging software (2 tumors per group, 10 fields per tumor were analyzed, p < .001). Two representative pictures from each group are shown.

Routine hematoxylin and eosin staining demonstrated that treatment of mice bearing orthotopic MiaPaCa-2 tumors with CT-322 resulted in a significant increase in tumor necrosis (Fig 6A). Furthermore, treatment with CT-322 induced a significant increase in the number of TUNEL-positive cells in the tumor tissue compared to control treated animals (Fig 6B).

## Discussion

Pancreatic cancer remains a deadly disease, with a dismal five year survival [1]. Contributing to the poor outcome is the resistance or insensitivity of most pancreatic cancers to chemotherapy. Gemcitabine, the current standard of care, extends life only minimally. Therefore, novel therapeutics agents are necessary to improve the survival associated with this disease.

Angiogenesis has been described as both a hallmark of cancer [2] and as an "organizing principle" around which anti-cancer therapy can be developed [20]. VEGF is a key mediator of tumor angiogenesis that functions primarily through VEGFR2. The complexity of the VEGF signaling pathway allows for multiple levels of inhibition, as seen by the large number of lead compounds entering trials that target this pathway [8,20]. One approach for VEGF inhibition is to reduce the availability of ligand (VEGF), as demonstrated by bevacizumab, a monoclonal antibody specific for human VEGF [9]. This approach has been validated in clinical trials combining chemotherapy and bevacizumab in the treatment of metastatic colorectal [10] or breast cancer [21]. However, despite initially promising results in pancreatic cancer, a subsequent randomized controlled trial failed to show clinical benefit of combining bevacizumab with gemcitabine [22,23].

In this series of experiments, we chose to investigate a novel anti-angiogenic agent. The Adnectin CT-322 binds VEGFR2 and prevents activation of the receptor. This approach is different than marketed tyrosine kinase inhibitors (e.g., sutent and sorafenib) in that CT-322 selectively blocks VEGFR2 activation without affecting other receptors or pathways. Additionally, the Adnectin compound utilizes *E. coli*-based manufacturing methods, making it well suited for clinical drug development.

The major finding of this study is that the adnectin CT-322 effectively controls the growth of orthotopic pancreatic tumors in mice by reducing VEGF-induced angiogenesis. We evaluated the efficacy of CT-322 in xenograft (MiaPaCa-2) and syngeneic (Pan02) tumors. As a single agent CT-322 appears to be more effective in the xenograft tumors where it reduced tumor growth by 50% whereas growth was reduced by less than 25% in the syngeneic model and was not different from tumors from control-treated animals. It is worth pointing out that Pan02 tumors are more invasive locally than MiaPaCa-2 tumors and often animals with Pan02 tumors need to be sacrificed due to disease burden that is far smaller than what is seen in xenograft models. For instance MiaPaCa-2 tumors from control-treated animals were on average over 1 g in size whereas Pan02 tumors from control-treated animals were on average just over 0.4 g in size. Thus it is possible that the xenograft tumors are more dependent upon VEGF-induced angiogenesis than the Pan02 tumors and thus anti-VEGF strategies are more effective as standalone agents in this setting.

We also observed in both tumor models that CT-322 as a single agent reduced microvessel density. Interestingly, Pan02 tumors from mice treated with CT-322 and gemcitabine did not show the same degree of reduction in microvessel density as tumors from mice treated with CT-322 alone. This is perplexing considering that the combination therapy was more effective at reducing overall tumor size. One possible explanation is that not all the blood vessels stained with MECA-32 are functional. In future studies this could determined by intravenous injection of an endothelial cell marker (eg., tomato lectin) or a fluorescent dye to track perfusion. An alternative non-exclusive explanation is that a high percentage of the blood vessels present in the tumors in the combination treated group have been co-opted from normal pancreas and are associated with pericytes and thus less sensitive to anti-VEGF therapy. This is plausible given that the size of the tumors is quite small in the combination treated group. It is worth pointing out that the tumor weights shown in figures 3 and 4 include residual normal pancreas, which typically weighs 0.1–0.15 g. It is difficult especially in the Pan02 model to dissect the tumor from the normal tissue due to the local invasion by the tumor. Thus, especially in the syngeneic model we may be underestimating the efficacy of CT-322-based therapy.

CT-322 has begun clinical trials and initial results from the first phase I studies were discussed recently at the 2008 American Society of Clinical Oncology meeting [24]. Thirty nine patients with solid tumors or non-Hodgkin's lymphoma were enrolled. 2 mg/kg/week was determined to be the maximum tolerated dose and out of 37 patients evaluated 49% had stable disease. These are promising results and have prompted a phase II study in combination with irinotecan in patients with recurrent glioblastome multiforme [24].

## Conclusion

Our results show that in two aggressive preclinical pancreatic cancer models, the strategy of selectively targeting VEGFR2 with CT-322 is effective. This compound is experimentally useful in that it blocks both mouse and human VEGFR2, thus allowing us to demonstrate the effectiveness of this drug in a fully immunocompetent mouse model. We also show that CT-322 enhances the efficacy of gemcitabine in this setting. As gemcitabine typically only prolongs life by a few months, agents improving its efficacy are needed. Further, these data demonstrate the safety and therapeutic utility of the Adnectin platform and suggest that further investigation of CT-322 is warranted in both animal models and clinical trials.

## Abbreviations

VEGF: Vascular endothelial growth factor; VEGFR1: VEGF receptor 1; VEGFR2: VEGF receptor 2; TCI: Tumor cell injection; PBS: Phosphate buffered saline; i.p. : Intraperitoneal; H&E: Hematoxylin and eosin.

## Competing interests

This study was supported in part by a sponsored research agreement from Adnexus to RA Brekken. Furthermore, R Mamluk and H Wong were full time employees at Adnexus at time of these studies.

Grant Support

This work was supported in part by the Effie Marie Cain Scholarship, a sponsored research agreement from Adnexus to RAB and a postdoctoral fellowship from the Susan G. Komen Foundation to SPD.

## Authors' contributions

SPD helped design some of the experiments, performed therapy studies, analyzed the data, and drafted the manuscript. LAS performed immunohistochemical analysis of tumor tissue, analyzed data, and participated in drafting the manuscript. AWB initiated the *in vivo *studies and helped design the experiments. AFM and JGC provided technical assistance throughout the study including all of the animal experiments and subsequent tissue analysis. RM conceived the study, provided material, assisted in the study design, and interpretation of the data. HW provided material, assisted in the interpretation of the data, and contributed to the drafting of the manuscript. RAB supervised and coordinated the study, finalized the manuscript, and obtained the funding to perform the studies. All authors read and approved the final manuscript.

## Pre-publication history

The pre-publication history for this paper can be accessed here:


